# Impact of initial dialysis modality on the survival of patients with ESRD in eastern China: a propensity-matched study

**DOI:** 10.1186/s12882-020-01909-3

**Published:** 2020-07-29

**Authors:** Xi Yao, Wenhua Lei, Nan Shi, Weiqiang Lin, Xiaoying Du, Ping Zhang, Jianghua Chen

**Affiliations:** 1 Kidney Disease Center, The First Affiliated Hospital, College of Medicine, Zhejiang University, No.79 Qingchun Road, Hangzhou, 310003 Zhejiang Province China; 2 Key Laboratory Of Nephropathy, Hangzhou, Zhejiang China

**Keywords:** End stage renal disease (ESRD), Hemodialysis, Peritoneal dialysis, Mortality

## Abstract

**Background:**

There are conflicting research results about the survival differences between hemodialysis(HD) and peritoneal dialysis (PD). The present study estimated the survival and the relative mortality hazard for incident HD and PD patients with end stage renal disease (ESRD) in eastern China.

**Methods:**

This study examined a cohort of patients with ESRD who initiated dialysis therapy in Zhejiang province between Jan of 2010 and Dec of 2014, followed up until the end of 2015. PD patients were matched in a 1:1 fashion with HD patients, and Kaplan–Meier analysis was used to explore the survival of them. The Cox proportional hazard regression model was applied to identify the factors that predict survival by treatment modality. Subgroup analyses were conducted by stratifying patients according to gender, age, causes of ESRD and comorbidities.

**Results:**

Among a total of 22,379 enrolled patients (17,029 HD patients and 5350 PD patients), 5350 matched pairs were identified, and followed for a median of 29 months (3 ~ 72 months). Kaplan-Meier survival curve revealed that overall mortality rate was significantly higher in HD patients than in PD patients (log-rank test, *P* < 0.001), after adjusting by gender, age, primary causes of ESRD and comorbidities. HD was consistently associated with an increased risk for morality compared with PD in the matched cohort (adjusted hazard ratio (AHR): 1.140, 95%CI: 1.023 ~ 1.271). In subgroup analyses, male, younger patients, or nondiabetic patients aged less than 65 years after adjustment of covariates, initiating with PD was associated with a significantly lower mortality compared with HD. In the multivariate Cox proportional risks model, age, diabetic nephropathy (DN), other/unknown causes of ESRD, and patients with a history of cardiovascular disease or cancer showed statistical significance in explaining survival of incident ESRD patients.

**Conclusions:**

ESRD patients who initiated dialysis with PD yielded superior survival rates compared to HD. Increased use of PD as initial dialysis modality in ESRD patients could be encouraged in Chinese population.

## Background

Renal replacement therapy (RRT) is a usual therapy for patients suffering from end stage renal disease (ESRD), including dialysis, either hemodialysis (HD) or peritoneal dialysis (PD) and kidney transplant [1, 2]. HD and PD are the most common choices of treatment in patients suffering from ESRD, due to the organ scarcity. A substantial body of evidence has been built around the outcomes of dialysis therapies, such as survival, health-related quality of life, and costs [3–5]. Among them, survival is one of the most significant outcomes, and in spite of the large number of studies, there is a considerable controversy about which dialysis modality provides a better survival. Comparisons of survival for patients on PD and HD, HD has been found to be associated with better survival [6–8], whereas, several studies indicate that PD patients have a better survival during the first 1 or 2 years [9, 10], and some recent studies infer that the survival of PD patients equates or even surpasses the survival of HD patients [11–14]. Survival can be attributed to the therapy itself or to other factors such as age, gender, diabetes mellitus (DM), history of cardiovascular disease (CVD), comorbidity at the start of therapy. However, publications on this subject for Asian populations are scarce, especially in Mainland China.

The number of patients with ESRD continues to increase in China, the prevalence rate of chronic kidney disease (CKD) in Mainland China is reported to 10.8, and 2% of them would progress to ESRD [15]. We established a Zhejiang Renal Disease System (ZJRDS) database in 2007, which collects demographic characteristics, comorbidity, dialysis clinical data, outcome-related data of dialysis patients, and distributes information on the incidence, prevalence, treatment, morbidity, and mortality of ESRD in Zhejiang province. Thus, comparisons of survival for incident HD and PD patients in Zhejiang province may represent the dialysis quality in eastern China.

Randomized controlled studies are the best to compare outcomes of different dialysis modalities, however, it is difficult to achieve them in clinical practice. The propensity score matching (PSM) is a statistical technique that can reduce bias resulting from the nonrandom nature of the treatment assignment seen in observational studies [16, 17]. Therefore, we conducted a study to describe and compare the mortality among incident HD and PD patients by using a propensity score–matched cohort.

## Methods

### Study design

This retrospective and observational cohort study included all incident ESRD patients on HD or PD from January 12,010 to December 31, 2014 in Zhejiang province, who had to be 18 years of age or older and had to have survived for the first 90 days on dialysis. All patients were followed until death, or switching to other renal replacement therapy (RRT), or December 31st, 2015 (the end of the study), after which survival data were censored. Patients were excluded if they had a history of kidney transplant, or no records in the dialysis start date and the initial dialysis modality, lacking demographic or clinical information. This study was approved by kidney disease center, the First Affiliated Hospital, College of Medicine, Zhejiang University. All patients allowed the usage of their clinical information, and all clinical investigations were conducted in accordance with the guidelines of Declaration of Helsinki.

### Study cohort

ZJRDS database, which was established for the purposes of improving dialysis quality in Zhejiang province, and includes from 254 hemodialysis centers and 101 peritoneal dialysis centers all over our province in 2019. We obtained all data from the ZJRDS database, which is privately owned by the Zhejiang dialysis quality control committee. Initial treatment modality was assigned as follows: patients starting on HD were assigned HD, whereas patients beginning with continuous ambulatory peritoneal dialysis (CAPD) or intermittent peritoneal dialysis (IPD) were classified as PD. Demographic and clinical information were collected upon entrance of patients into the cohort, including gender, age, primary causes of the ESRD (chronic glomerulonephritis (CGN), DM, hypertension, polycystic kidney disease (PKD), others or unknown causes), vascular access types and comorbidities. Comorbidities included a history of CVD (coronary artery disease, arrhythmia, congestive heart failure, peripheral vascular disease and cerebrovascular disease), chronic obstructive pulmonary disease (COPD), gastrointestinal ulcer, moderate to severe chronic liver disease and malignancy. All patients were follow-up to December 31st, 2015, or until the occurrence of the death or censorship for all those events in which the patient was alive but could not conclude the follow-up period, including kidney transplantation, loss to follow-up and change of dialysis modality.

### Statistical analyses

To address the imbalance of the effects of gender, age, causes of ESRD and comorbidity, we matched PD group with HD group using propensity scores with a one-to-one nearest neighbor caliper width of 0.02. We calculated the propensity score for PD and HD patients using a logistic regression model to estimate the probability of the dialysis modality on the basis of baseline variables such as age, gender, cause of the ESRD, diabetes, history of CVD, COPD, gastrointestinal ulcer, chronic liver disease and malignancy. In both the baseline and the matched cohorts, continuous variables are expressed as mean ± SD for normally distributed data, or as median and frequency (%) for non-normally distributed data, differences in patients’ characteristics between HD and PD group were analyzed by t-test or the Mann-Whitney tests for continuous variables, whereas the χ^2^ test was used for categorical variables [18, 19]. In both the entire cohort and the matched cohort, we constructed Kaplan-Meier curves for all-cause mortality. The risk of all-cause mortality for HD patients compared with PD patients was estimated as adjusted hazard ratios (HRs) with 95% CIs by using the multivariable Cox proportional hazards model adjusted for gender, age, sex, cause of the ESRD and comorbidity.

All statistical analyses were conducted using the SPSS (Statistical Package for Social Sciences) 22.0 software (IBM, Armonk, NY, USA) with a R software -plug-ins (R-2.15.3, https://cran.r-project.org/bin/windows/base/old/) [20, 21]. Statistical tests were considered significant at *P* < 0.05 (two-sided).

## Results

### Characteristics of the patients

**Fig. 1:**
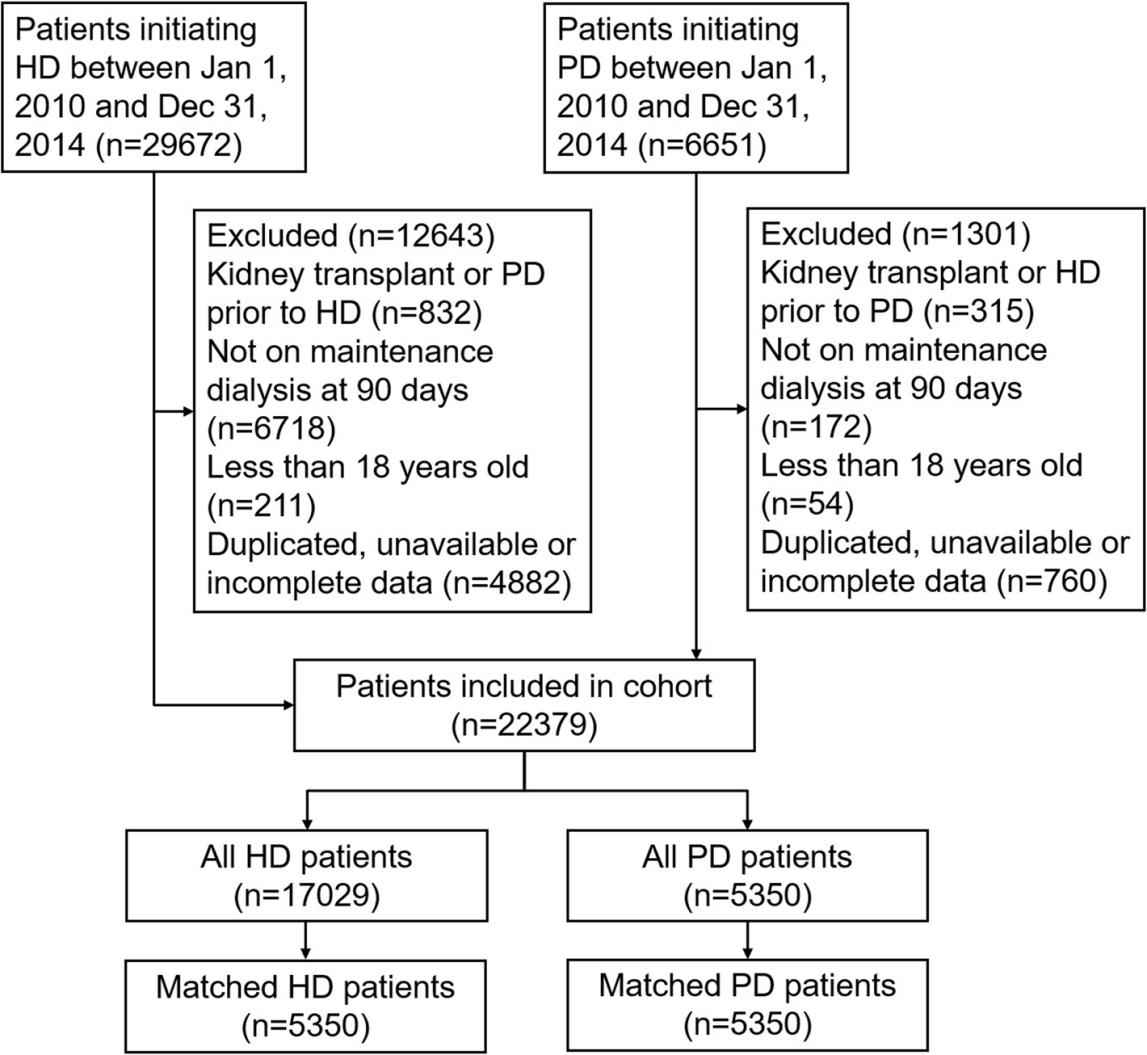
Derivation of the whole cohort

Between Jan of 2010 and Dec of 2014, 36,323 patients initiated dialysis in Zhejiang province. 13,944 patients were excluded, the derivation of the whole cohort is detailed in Fig. 1. (Derivation of the whole cohort). A total of 22,379 ESRD incident patients (17,029 (76.1%) HD and 5350 (23.9%) PD patients) were enrolled in our analysis, who were followed for a median of 29 months (range, 3 ~ 72 months). The study subjects were more likely to be male (57.9%) and young (63.8% patients was under the age of 65 years old). Patients with diabetes accounted for 23.2%, and 929 (4.2%) patients had malignancy. 770 (3.5%) patients switched modality, including 258 (1.2%) HD switched to PD and 512 (2.3%) PD switched to HD, 838 (3.7%) patients were transplanted, and 2061 (9.2%) were at lost during the following period. A comparison of demographic and clinical characteristics between the HD and PD groups showed that, on average, patients in the PD group were significantly younger (53 ± 15 years in PD versus 58 ± 16 in HD, *p* < 0.001); had a significantly lower proportion of males (53.6% in PD versus 59.3% in HD, *p* < 0.001), a history of CVD (17.7% versus 21.2%, *p* < 0.001), malignancy (1.7% versus 4.3%, *p* < 0.001) and COPD (0.4% versus 0.8%, *p* < 0.05). In terms of the ESRD etiology, the PD group had a lower proportion of patients having diabetes as the leading cause (8.7% in PD versus 20.7% in HD, *p* < 0.001), and PKD as the leading cause (2.4% versus 4.5%, *p* < 0.001) (Table 1.).

**Table 1:** Baseline characteristics of 17,029 hemodialysis (HD) and 5350 peritoneal dialysis (PD) patients

Baseline Characteristics	HD(***n*** = 17,029)	PD(***n*** = 5350)	***P***-value	Standardized differences
Male,n(%)	10,096 (59.3)	2868 (53.6)	< 0.001	0.115
Age (years)	58 ± 16	53 ± 15	< 0.001	0.322
65+ years,n(%)	6827 (40.1)	1276 (23.9)	< 0.001	0.353
**Age group,n(%)**			< 0.001	
18 ~ 49 years	5355 (31.4)	2343 (43.8)		0.258
50 ~ 59 years	3370 (19.8)	1277 (23.9)		0.099
60 ~ 69 years	3610 (21.2)	1002 (18.7)		0.063
70+ years	4694 (27.6)	728 (13.6)		0.351
**Causes of ESRD,n(%)**				
CGN,n(%)	8037 (47.2)	2527 (47.2)	0.962	0.000
DN,n(%)	3523 (20.7)	468 (8.7)	< 0.001	0.344
HTN,n(%)	1343 (7.9)	440 (8.2)	0.426	0.251
PKD,n(%)	766 (4.5)	126 (2.4)	< 0.001	0.115
Other/Unknown,n(%)	3360 (19.7)	1789 (33.4)	< 0.001	0.314
**Comorbid conditions,n(%)**				
DM,n(%)	958 (5.6)	270 (5.0)	0.105	0.027
CVD,n(%)	3606 (21.2)	949 (17.7)	< 0.001	0.089
Malignancy,n(%)	734 (4.3)	90 (1.7)	< 0.001	0.153
Chronic liver disease,n(%)	870 (5.1)	267 (5.0)	0.731	0.005
COPD,n(%)	128 (0.8)	20 (0.4)	< 0.05	0.052
Gastrointestinal ulcer,n(%)	90 (0.5)	28 (0.5)	0.964	0.000

We matched 5350 pairs of patients by propensity scores, based on patients’ age, gender, primary causes of ESRD and comorbidities. 11,679 HD patients were excluded, who were significantly older (61 ± 16 years in excluded HD versus 53 ± 15 in included HD, *p* < 0.001); had a significantly higher proportion of males (61.5% versus 54.5%, *p* < 0.001), diabetes (6.0% versus 4.9%, *p* < 0.05), a history of CVD (22.9% versus 17.4%, *p* < 0.001), malignancy (5.6% versus 1.6%, *p* < 0.001) and COPD (0.9% versus 0.4%, *p* < 0.05). In terms of the ESRD etiology, the excluded HD group had a higher proportion of patients having diabetes as the leading cause (26.1% versus 8.9%, *p* < 0.001), and PKD as the leading cause (5.5% versus 2.3%, *p* < 0.001) (Supplemental Table 1.) The basal characteristics were different between HD patients and PD patients. Baseline characteristics for the propensity scores - matched cohorts are detailed in Table 2. As expected, baseline characteristics were well balanced between HD and PD patients in the matched cohort.

**Table 2:** Baseline characteristics for the propensity score–matched cohort

Baseline Characteristics	HD(***n*** = 5350)	PD(***n*** = 5350)	***P***-value
Gender,n(%)	2916 (54.5)	2868 (53.6)	0.352
Age (years)	53 ± 15	53 ± 15	0.874
65+ years,n(%)	1283 (24.0)	1276 (23.9)	0.874
**Age group,n(%)**			0.4
18 ~ 49 years	2352 (44.0)	2343 (43.8)	
50 ~ 59 years	1261 (23.6)	1277 (23.9)	
60 ~ 69 years	957 (17.9)	1002 (18.7)	
70+ years	780 (14.6)	728 (13.6)	
**Causes of ESRD,n(%)**			
CGN,n(%)	2541 (47.5)	2527 (47.2)	0.786
DN,n(%)	477 (8.9)	468 (8.7)	0.759
HTN,n(%)	438 (8.2)	440 (8.2)	0.944
PKD,n(%)	125 (2.3)	126 (2.4)	0.949
Other/Unknown,n(%)	1769 (33.1)	1789 (33.4)	0.682
**Comorbid conditions,n(%)**			
DM,n(%)	260 (4.9)	270 (5.0)	0.656
CVD,n(%)	931 (17.4)	949 (17.7)	0.647
Malignancy,n(%)	85 (1.6)	90 (1.7)	0.703
Chronic liver disease,n(%)	268 (5.0)	267 (5.0)	0.965
COPD,n(%)	22 (0.4)	20 (0.4)	0.757
Gastrointestinal ulcer,n(%)	24 (0.4)	28 (0.5)	0.578

### Comparisons of mortality according to dialysis modalities

**Fig. 2:**
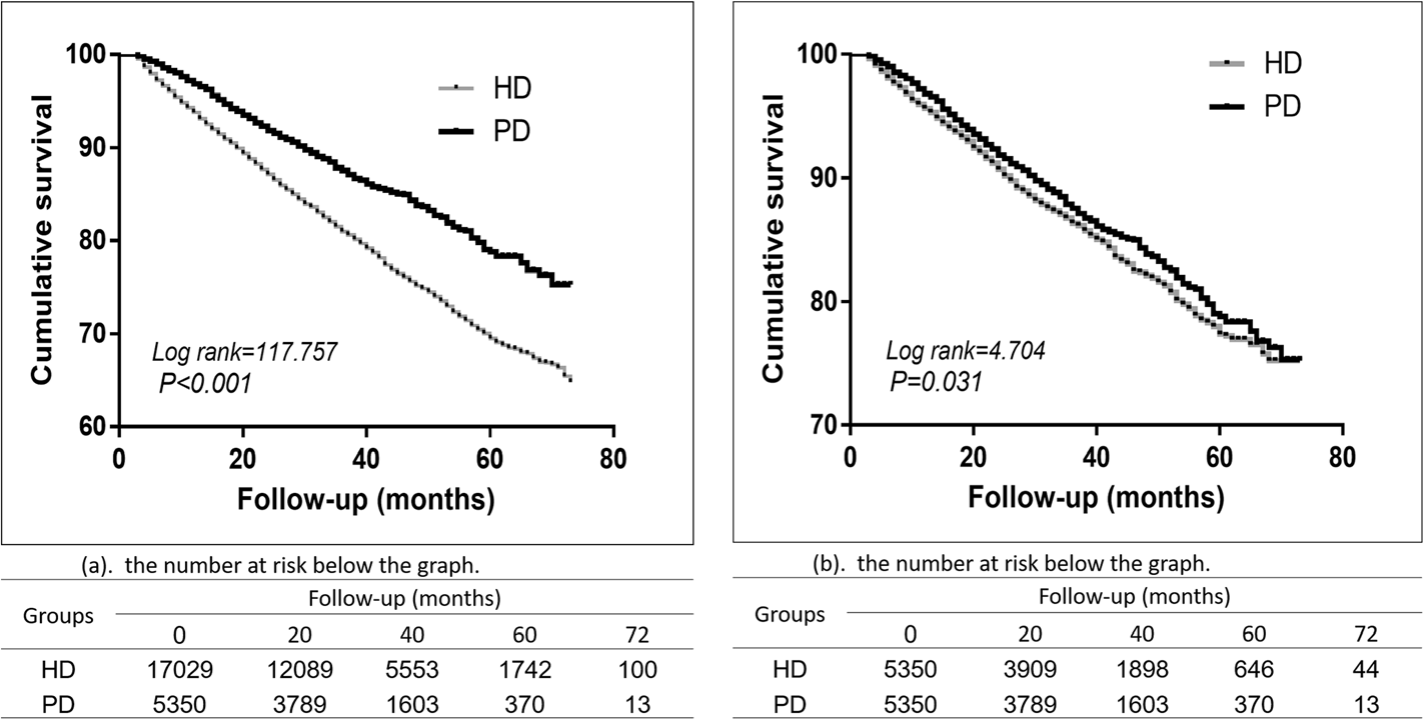
**a** Kaplan–Meier survival curve according to the initial dialysis modality (whole cohort); log-rank test, *P* < 0.001. **b** Kaplan–Meier survival curve according to the initial dialysis modality (matched cohort); log-rank test, *P* = 0.031

During the follow-up period, 3182 patients undergoing HD (18.7%) and 602 patients undergoing PD (11.3%) died. The overall 1-, 2-, 3-, 4-, and 5-year survival rates for HD and PD patients both in whole cohort and matched cohorts are given in Table 3. By the Kaplan–Meier analysis with log-rank test, statistically significant differences were found in patient survival by therapy, with a better survival for PD patients compared to HD patients (Fig. 2a. Kaplan–Meier survival curve according to the initial dialysis modality (whole cohort); log-rank test, *P* < 0.001). Using the Cox proportional hazard model adjusting by age, gender, causes of ESRD and comorbid conditions showed that PD was superior to HD as an initial modality in maintenance dialysis (HD vs PD AHR: 1.239, 95% confidence interval (CI):1.130–1.358, *P* < 0.001).

**Table 3:** Survival rate among ESRD patients by follow-up year in the whole cohort and propensity score-matched corhort

Follow-up duration,year	Whole Cohort		Macthed Cohort	
	HD(%)	PD(%)	HD(%)	PD(%)
**1**	93.4	96.6	95.4	96.6
**2**	86.8	91.5	90.4	91.5
**3**	80.6	87.2	86.1	87.2
**4**	74.7	83.4	81.8	83.4
**5**	69.4	78.4	77.1	78.4

In the matched cohorts with Kaplan–Meier plots, survival in PD patients was still better than that in HD patients (Fig. 2b. Kaplan–Meier survival curve according to the initial dialysis modality (matched cohort); log-rank test, *P* = 0.031). Cox proportional hazard model adjusting by covariates revealed that better survival seen in PD group compared with the HD group (HD vs PD AHR: 1.140; 95% CI: 1.023–1.271, *P* < 0.05).

Demographic characteristics, comorbidity were analyzed using Cox proportional hazard models (forward stepwise, probability remove: 0.1) to identify independent mortality risk factors. Table 4 presents a summary of analytical results, HD (vs. PD) was associated with an increased risk for morality with an AHR of 1.140 (95%CI: 1.023–1.271). Age, diabetic nephropathy (DN), other/unknown causes of ESRD (including lupus nephritis, interstitial nephritis, Hepatitis B Virus associated glomerulonephritis and so on), and patients with comorbid conditions, such as a history of diabetes, CVD or malignancy had increased overall mortality (Table 4.).

**Table 4:** Risk for all-cause mortality using matched cohort

Characteristics	β	AHR	95%CI	***P***-value
HD:PD	.131	1.140	1.023 ~ 1.271	< 0.05
Age	.049	1.050	1.046 ~ 1.055	< 0.001
**Causes of ESRD**				< 0.001
DN	.738	2.091	1.774 ~ 2.465	< 0.001
HTN	.185	1.203	0.998 ~ 1.45	.052
PKD	−.134	.874	0.575 ~ 1.329	.529
Other/Unknown	.371	1.449	1.274 ~ 1.65	< 0.001
**Comorbid conditions**				< 0.001
DM	.283	1.328	1.084 ~ 1.626	< 0.05
CVD	.221	1.247	1.099 ~ 1.415	< 0.001
Malignancy	1.301	3.673	2.893 ~ 4.663	< 0.001

Multivariable Cox proportional hazards model, adjusted for age, gender, cause of the ESRD, diabetes, history of cardiocerebral vascular diseases, COPD, gastrointestinal ulcer, chronic liver disease and malignancy

### Subgroup analyses according to baseline covariates

**Fig. 3:**
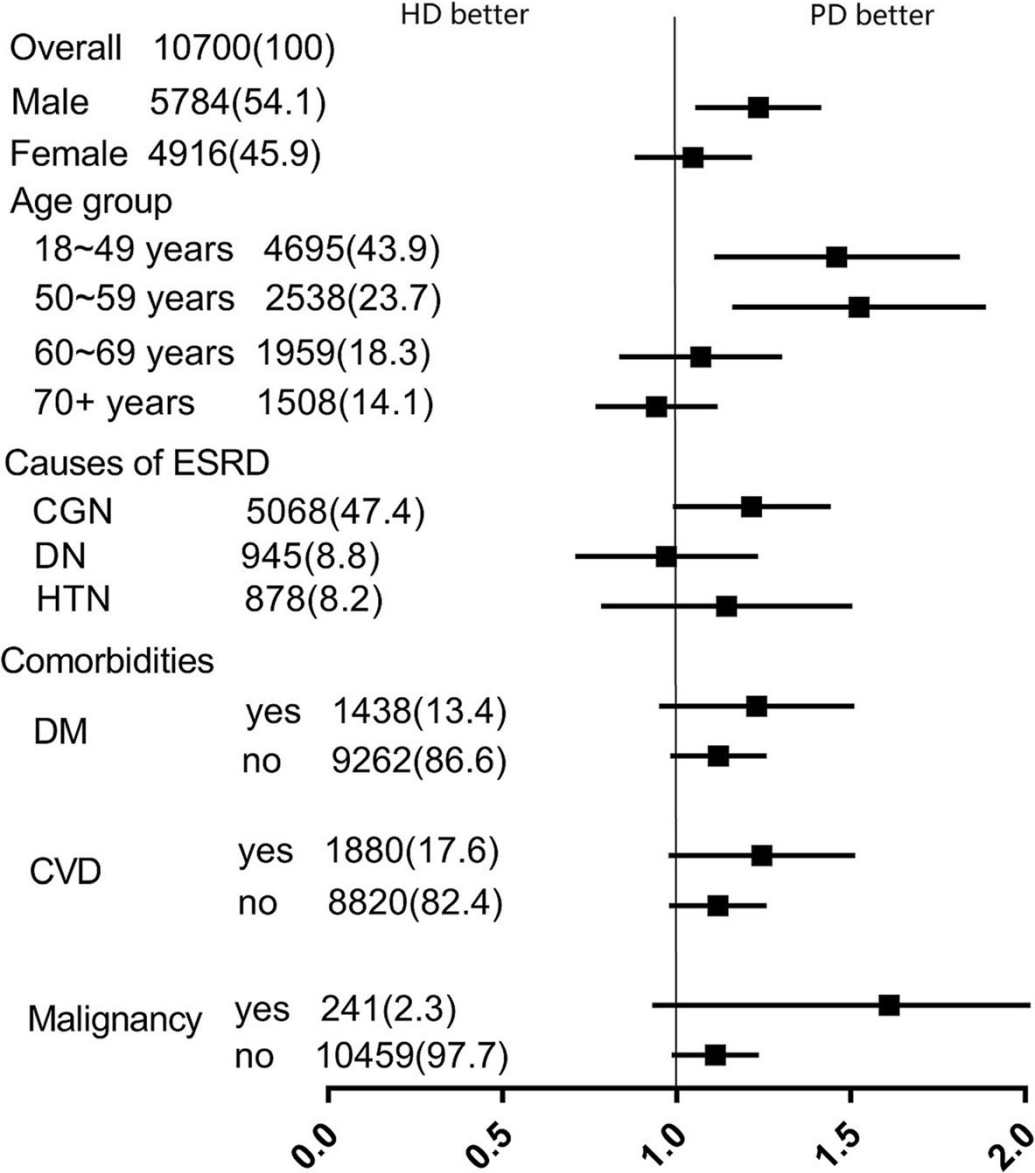
Risk for all-cause mortality associated with initial dialysis modality for difference subgroups (matched cohort)

**Fig. 4:**
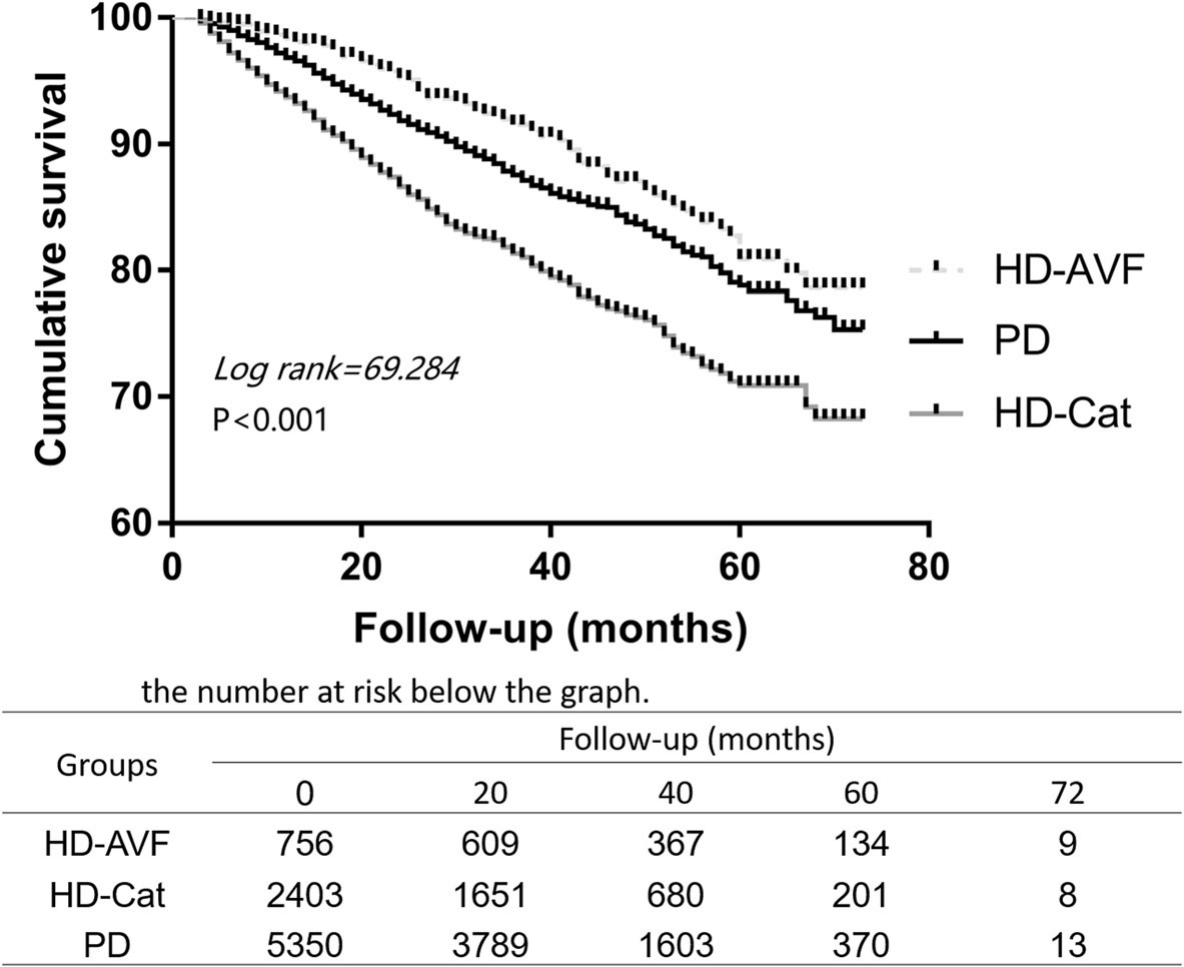
Kaplan-Meier survival curves for all-cause mortality for HD patients with arteriovenous fistula (AVF) or catheters (Cat), versus PD patients (matched cohort)

We stratified the matched cohort into subgroups according to various baseline covariates. As shown in Fig. 3 (Risk for all-cause mortality associated with initial dialysis modality for difference subgroups (matched cohort)), this increased mortality risk associated with HD was constant across some subgroups (male and younger age). HD patients with arteriovenous fistula (AVF) had a similar survival rate with PD patients, but HD patients with catheters had a poor survival outcome (Fig. 4. Kaplan-Meier survival curves for all-cause mortality for HD patients with arteriovenous fistula (AVF) or catheters (Cat), versus PD patients (matched cohort)).

**Fig. 5:**
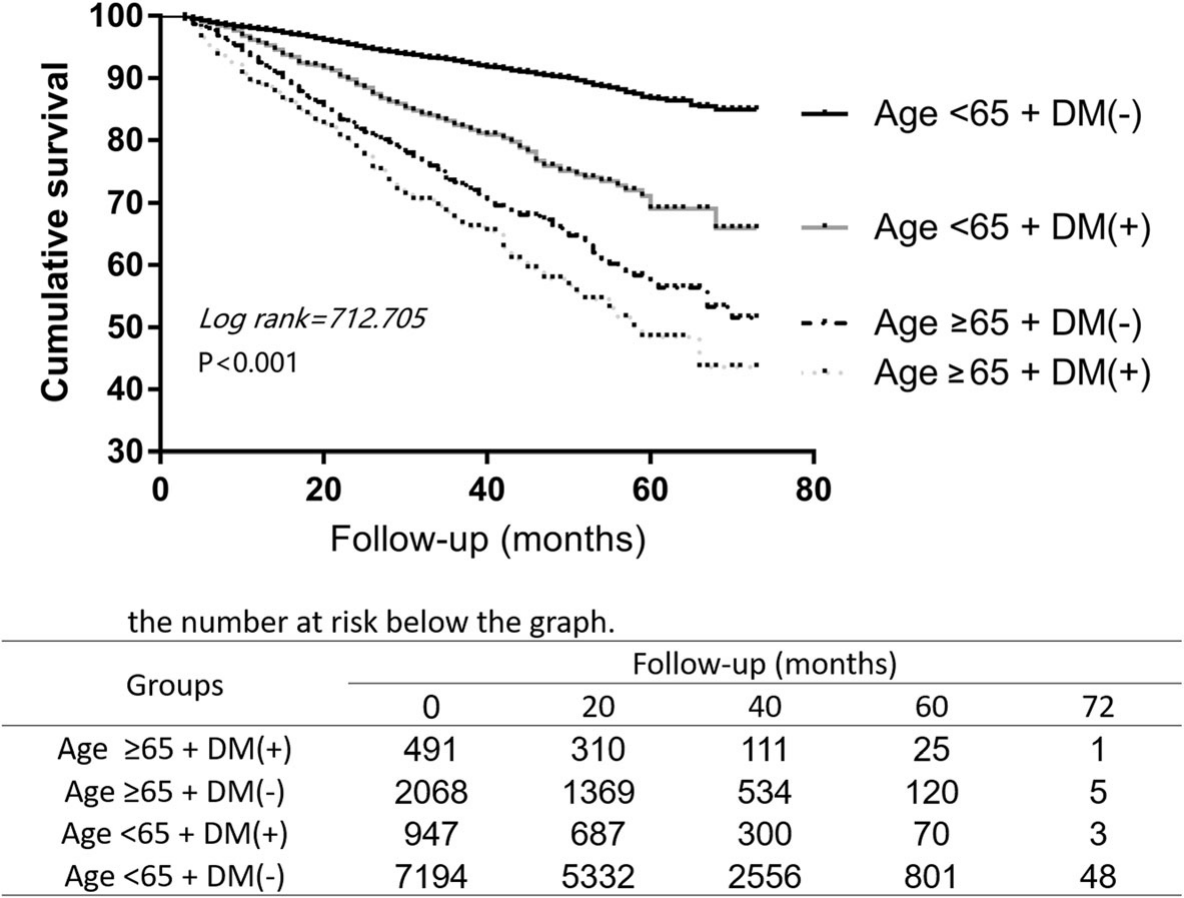
Kaplan–Meier survival curve according to the initial dialysis modality and diabetes mellitus (DM) (matched cohort)

**Fig. 6:**
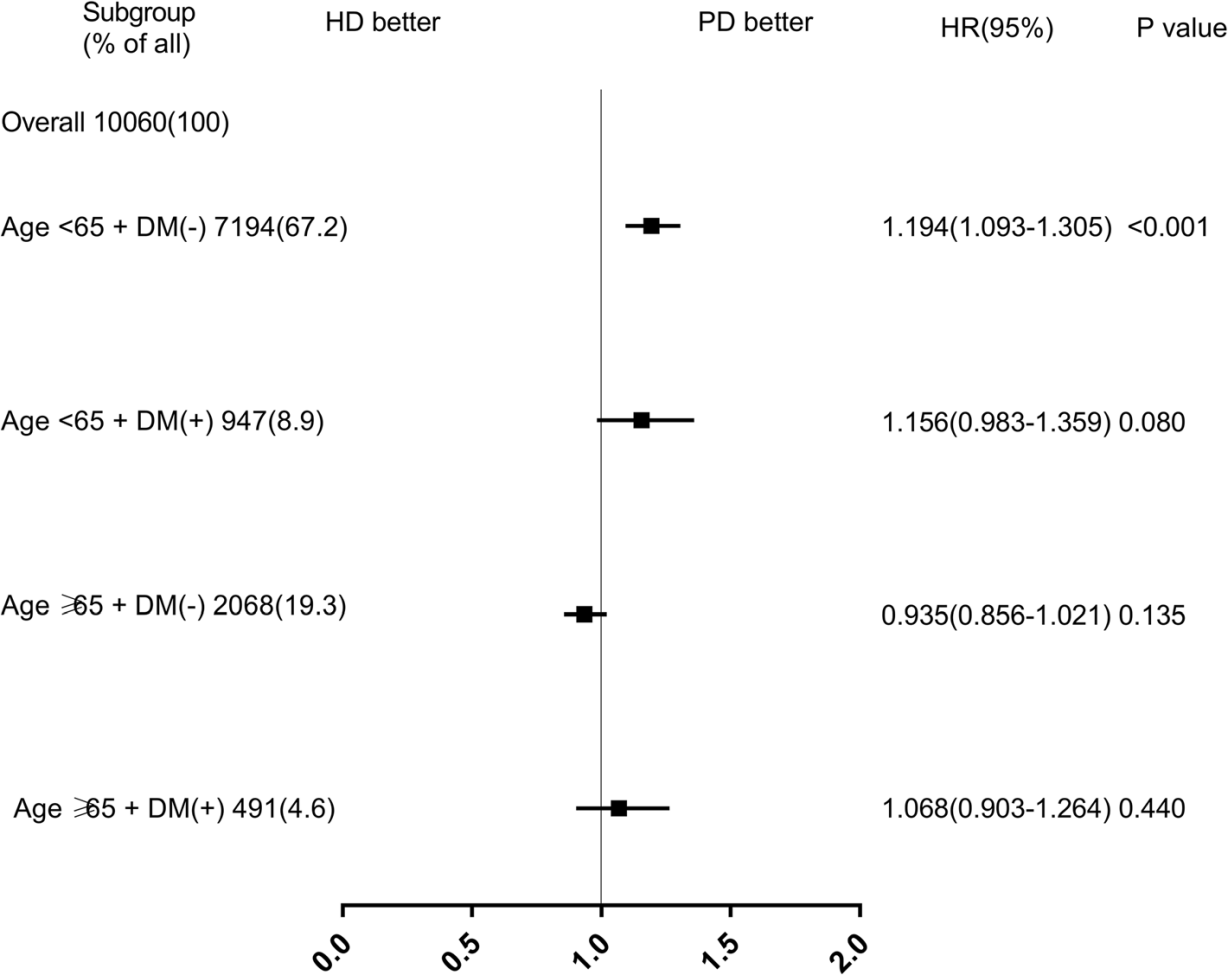
Risk for all-cause mortality associated with initial dialysis modality for diabetes subgroups (matched cohort)

Patients with or without DM did not show statistically significant differences in survival between HD and PD group. Then, we further stratified them into younger (< 65 years old) and older group (≥65 years old). Kaplan–Meier method with log-rank test revealed that elderly patients with DM were associated with lower survival (Fig. 5. Kaplan–Meier survival curve according to the initial dialysis modality and diabetes mellitus (DM) (matched cohort)), the Cox proportional hazard model showed that the survival of nondiabetic patients younger than 65 years subgroup was better on PD (HD vs PD HR: 1.194, 95%CI: 1.093 ~ 1.305, *P* < 0.001) (Fig. 6. Risk for all-cause mortality associated with initial dialysis modality for diabetes subgroups (matched cohort)).

## Discussion

In this study of ESRD patients initiating dialysis, the all-cause mortality was higher in HD patients than that in PD patients in the whole cohort and propensity score–matched cohort. In the adjusted Cox model of time to mortality of the matched cohort, age, DN, other/unknown causes of ESRD, history of DM, CVD and malignancy were independent predictors of mortality. To our knowledge, this is the first large-scale study that uses PSM in a survival comparison of incident PD patients versus HD patients in China.

Our study demonstrated that HD was associated with an increased risk for all-cause mortality compared with PD in the matched cohort (HR 1.140, 95%CI: 1.023 ~ 1.271), which is similar with some previous researches from many countries, such as the United States, India and Korea [22–24]. The survival benefit of PD over HD is obvious in the first 1–2 years of dialysis treatment. Researches from Canada and Denmark showed that PD patients had a lower mortality than HD patients in the first 2 years after initiation of dialysis [10, 25]. There are several reasons to explain why ESRD patients favorite PD as the initiating therapy. Earlier studies are typically based on the late 1990s and early 2000s, and technological progress of HD and PD has accelerated during the last decades, especially after the “PD First” policy innovated into PD care [26], which makes tremendous contributions to the PD patients’ longevity. The apparent survival advantage of PD may also be due to a lower co-morbidity and a lower burden of acute onset ESRD at the inception of dialysis. In our study, HD patients were older, had more comorbidities than PD patients at the enrollment, whom inclined to choose urgent-start HD in order to balance their inner environment as soon as possible. Urgent-start HD patients with central catheter as an initial vascular access had a relation with higher mortality [27], which could promote vascular endothelial damage, lead to inflammatory reaction and increase the chance for infection. Garcia-Canton C, et al. had revealed that HD patients with central catheter had the lowest survival rate, however, patients with AVF had a comparable survival rate with PD patient [28], which was also observed in our study. Compared with HD, PD has a smaller effect on hemodynamics and fewer dietary restrictions [29], could protect the residual renal function, which is an independent protective factor in survival of ESRD patients [30]. Moreover, when PD patients have technique fail, it is more common for them to switch to HD, therefore their mortality rate was lower than HD patients.

In a subgroup analysis of our matched cohort, male or younger patients of initiating with PD exhibited a significant better survival versus those initiating with HD. This changing may be due to PD technique is relatively easy and has a lower prevalence of preexisting CVD at the initiation of dialysis therapy at a younger age. Particularly in nondiabetic patients aged less than 65 years after adjustment of covariates, initiating with PD was associated with a significantly lower mortality compared with HD. This finding is similar to results from Netherlands, USA, Columbia and Korea, which reported a lower mortality in younger nondiabetic ESRD patients initiating with PD versus HD [22, 24, 31, 32]. Previous studies suggested that PD patients were with higher blood lipid level than HD patients, which might accelerate the process of arteriosclerosis and increase incidences of cardiovascular events [33]. In addition, diabetic patients are easy to be involved with disorders of lipid metabolism [34, 35], and PD therapy may affect blood glucose of ESRD patients [36, 37]. Thus, PD was associated with significant higher survival compared with HD in younger without diabetic subgroup.

This study has several limitations worth mentioning. First, this is an retrospective observational study and PSM can only account for observed confounders without any account for unobserved confounders. Therefore, this study may not be completely free of bias due to confounding, because the initial RRT was not randomly allocated, causality cannot be demonstrated as in the experimental design of a randomized controlled study. Second, we stopped follow-up till the occurrence of censorship for those events in which the patient was alive but could not conclude the follow-up period, which includes kidney transplant and change of dialysis modality. The effect of switching the type of RRT in time-dependent models and more patients switched from PD to HD than from HD to PD was not considered. Third, we could not ascertain the severity of comorbidities because the data were extracted from a database, for example, we cannot calculate the Charlson Comorbidity Index (CCI) in our study. Fourth, this is a retrospective study, several important laboratory characteristics are not available in the database, such as residual renal function, Kt/V and iPTH.

Despite these limitations, strengths of our study include the large study population that allowed us to assemble the largest PSM cohort of Chinese patients with ESRD initiating with either PD or HD. And this study may have clinically relevant features and may help doctors and patients to make proper dialytic modality choices in Zhejiang province.

## Conclusions

ESRD patients who initiated dialysis with PD yielded superior survival rates compared to HD. Thus, increased use of PD should be given greater consideration when initiating RRT in Chinese population.

## Supplementary information

**Additional file 1: Table S1.** Baseline characteristics of 7761 excluded hemodialysis (HD) and 541 excluded peritoneal dialysis (PD) patients.

## Data Availability

Data were from Zhejiang Renal Disease System (ZJRDS) database (http://zjdialysis.com/ZK/web-index!showIndex.action).

## Abbreviations

PD: Peritoneal dialysis
HD: Hemodialysis
ESRD: End stage renal disease
PSM: Propensity score matching
ZJRDS: Zhejiang Renal Disease System
CKD: Chronic kidney disease
RRT: Renal replacement therapy
CVD: Cardiovascular diseases
COPD: Chronic obstructive pulmonary disease
HR: Hazard ratio
CI: Confidence interval
AVF: Arteriovenous fistula
CAPD: Continuous ambulatory peritoneal dialysis
IPD: Intermittent peritoneal dialysis
DM: Diabetes mellitus
DN: Diabetic nephropathy
HTN: Hypertensive nephropathy
CGN: Chronic glomerulonephritis
PKD: Polycystic kidney disease
